# Respiratory symptoms among e-cigarette users without an established smoking history in the VERITAS cohort

**DOI:** 10.1038/s41598-024-80221-8

**Published:** 2024-11-18

**Authors:** Jefrrey Zamora Goicoechea, Allison Boughner, Juan José Cirion Lee, Aman Mahajan, Kurt Yeo, Maris Sproga, Christopher Russell, Michael Coughlan, Arielle Selya, Grazia Caci, Pasquale Caponnetto, Venera Tomaselli, Riccardo Polosa

**Affiliations:** 1 International Network of Nicotine Consumer Organisations (INNCO), Vejle, Denmark; 2 Asociación de Reducción de Daños del Tabaquismo (ARDT Iberoamérica), Bogota, Colombia; 3 Asociación de usuarios de vaporizadores y métodos de reducción de daños por tabaquismo de Costa Rica (ASOVAPE), San José, Costa Rica; 4 American Vapor Manufacturers, Prescott, AZ USA; 5 South Carolina Vapor Association, Charleston, SC USA; 6 México y el Mundo Vapeando, Mexico City, Mexico; 7 Independent THR Activist, New Delhi, India; 8 Vaping Saved My Life, Benoni, Gauteng South Africa; 9 World Vapers Alliance, Miami, FL USA; 10 Independent THR Activist, Riga, Latvia; 11 Russell Burnett Research & Consultancy Ltd., Glasgow, UK; 12 ECLAT Srl, Spin-off of the University of Catania, Catania, Italy; 13 Pinney Associates, Inc., Pittsburgh, PA USA; 14 UOC MCAU, University Teaching Hospital “Policlinico-S.Marco”, University of Catania, Catania, Italy; 15 Department of Science of Education, Section of Psychology, University of Catania, Catania, Italy; 16 Center of Excellence for the Acceleration of HArm Reduction (CoEHAR), University of Catania, Via S. Sofia, 78—Ed. 4, p. 2, 95123 Catania, Italy; 17 Department of Economics and Business, University of Catania, Catania, Italy; 18 Department of Clinical & Experimental Medicine, University of Catania, Catania, Italy; 19 Faculty of Medicine and Surgery, “Kore” University of Enna, 94100 Enna, Italy

**Keywords:** Electronic cigarettes, Health effects, Respiratory symptoms, Survey, Real-world use study, Diseases, Signs and symptoms

## Abstract

Prior research on e-cigarettes’ health impacts is inconclusive due to confounding by previous tobacco smoking. Studies of e-cigarette use among people without an established smoking history are informative for this question. A cross-sectional survey was administered across six geopolitical world regions to adults aged 18+ without a history of established cigarette smoking or regular use of other nicotine/tobacco products. Two cohorts were defined based on e-cigarette use: “Vapers Cohort” (N = 491) who used e-cigarettes in the past 7 days and “Control Cohort” (N = 247) who never regularly used e-cigarettes. Frequency of respiratory symptoms (Respiratory Symptom Evaluation Score (RSES)) were compared between cohorts, adjusting for sociodemographics. Tobacco use history and patterns of e-cigarette use was also examined. Respiratory symptoms were rare among both the Vapers and Control Cohorts: 83.3% and 88.4%, respectively, reported “rarely” or “never” experiencing all five RSES items (*p* = 0.125). The Vapers (vs. Control) Cohort reported modestly more frequent respiratory symptoms (adjusted mean RSES 1.61 vs. 1.43, respectively, *p* < 0.001); however, this difference (0.18) did not reach the threshold of clinical relevance (0.57). The Vapers (vs. Control) Cohort more often reported former cigarette experimentation (30.8% vs. 12.1%) and former infrequent use of other nicotine/tobacco products (18.1% vs. 5.8%). The Vapers Cohort most often used disposable devices (63.7%) and multiple flavors (approximately 70–80% across primary device type). In this cohort of adults without a history of established combustible tobacco use, e-cigarette use was statistically linked to more frequent respiratory symptoms, though not in a clinically meaningful way. The cross-sectional design of this study cannot establish causality between e-cigarette use and respiratory symptoms.

## Introduction

Electronic cigarettes (ECs) have become increasingly popular among people who smoke (PWS) as a potential harm reduction tool and smoking cessation aid^1,2^ due to their cost-effectiveness^3^ and the ability to mimic the smoking experience without combustion or smoke production^4,5^.

However, concerns have been raised about the health risks of ECs, with several papers reporting higher rates of respiratory conditions among people who use ECs than among nonusers^6–8^. However, since the majority of adults who use ECs currently or formerly smoke cigarettes^9^, this apparent association is at least partly confounded by cigarette smoking history.

Evidence for direct harms that are uniquely attributable to EC use is lacking. Under typical use conditions, without the overheating of coils or dry burning, ECs produce significantly lower exposures to harmful substances than do tobacco cigarettes^6–8,10–12^. In the US, the outbreak of e-cigarette or vaping-associated lung injury (EVALI) was initially attributed to nicotine e-cigarettes, but the true cause was later identified as a cutting agent—vitamin E acetate—in illicit cannabis vapes^13,14^. No definitive respiratory health effects *uniquely* attributable to vaping have been conclusively proven^10,12,15,16^.

The prevalence of e-cigarette use among non-smokers remains largely unexplored and depends heavily on data quality. Reliable evidence primarily comes from well-funded studies in the US and UK. For instance, the US NHIS 2021 reported that 2.9% of never-smokers currently use e-cigarettes^17^, while the 2024 ASH report from Great Britain indicated a prevalence of 1.6% among never-smokers^18^. A pooled analysis of 15 countries from the Global Adult Tobacco Survey (GATS) shows that vaping is rare among adults who have never smoked, with only 0.1% of never-smokers currently using e-cigarettes^19^. However, some LMIC studies present an unclear picture, often due to sample issues. For example, a 2023 survey in Ecuador found that 44.2% of adult e-cigarette users had never smoked, though this sample was skewed towards healthcare workers^20^. Similarly, a 2019 Brazilian survey showed most e-cigarette users had never smoked, though usage was more common among younger adults^21^.

Given that existing reports of respiratory effects attributable to ECs are likely confounded by history of combustible tobacco use^15,22,23^, research on people *without* an established smoking history can be especially informative about whether vaping may have adverse respiratory effects. In a preliminary prospective study, daily users of ECs without a history of established smoking showed no significant alterations in lung function, respiratory symptoms, or exhaled breath nitric oxide (eNO)^16,24^. Furthermore, high-resolution computed tomography (HRCT) scans revealed no significant structural abnormalities in the lungs over an average observation period of 3.5 years^16,24^. However, the study faced limitations, such as a small sample size that may have included healthier e-cigarette users, and relatively short follow-up duration (3.5 years).

The current **V**aping **E**ffects: **R**eal-world **I**n**T**ern**A**tional **S**urveillance (VERITAS) Study aims to fill this knowledge gap about long-term effects of exclusive vaping on lung health. To this end, the VERITAS study sampled adults without a history of established smoking or regular use of other tobacco/nicotine products, and compared frequency of respiratory symptoms among those who used ECs and those who did not. This study also evaluated the feasibility of gathering a large, global, distinct cohort of EC users for this and future research, and characterized these EC users’ patterns of use.

## Methods

**Fig. 1:**
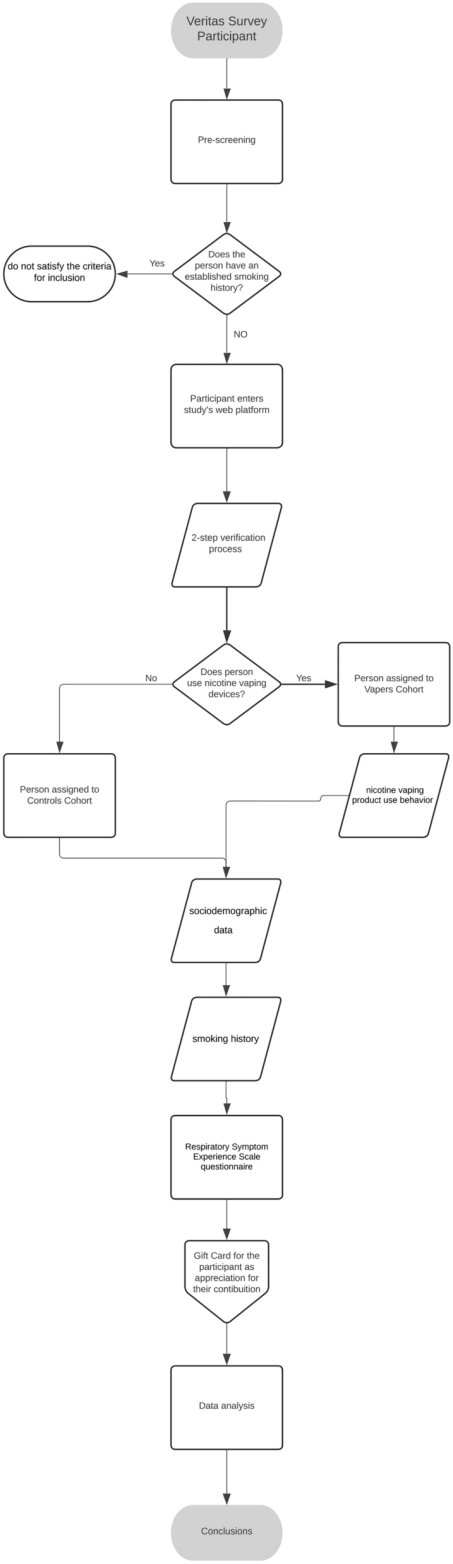
The algorithm illustrates the sequential steps of the VERITAS survey process. Participants undergo pre-screening to determine their smoking history. Those with an established smoking history are excluded. Eligible participants are categorized into two cohorts: vapers (those who use vaping devices) and controls (non-users). Data collection includes sociodemographic information, smoking history, nicotine vaping product use behavior, and responses to the Respiratory Symptom Experience Scale (RSES) questionnaire.

The outline of the study is illustrated in Fig. 1.

### Study design

The protocol of the VERITAS study has been described in detail previously^25^. Briefly, this study was a cross-sectional, internet-based survey conducted in six geopolitical world regions (Africa & Middle East, North America, Latin America & South America, Asia–Pacific, Western Europe, Eastern Europe). The study protocol received approval from the Ethics Review Board of the Dipartimento di Scienze della Formazione Sezione di Psicologia at the University of Catania (approval date, 25 November 2020). Data collection occurred between 10 May 2023 and 26 November 2023. All methods were performed in accordance with the relevant guidelines and regulations.

### Participants

Based on sample size calculations and power analysis (see Supplement), this study targeted enrollment of 750 participants total (targets: Vapers Cohort = 500; Control Cohort = 250), approximately equally across regions.

This study recruited individuals 18 years and older who met the following criteria:

1. Vapers Cohort (N = 491):
  - Past 7-day use of at least one of three types of electronic cigarettes/vapes (disposables, rechargeable with replaceable pre-filled pods or cartridges (“pod/cartridge devices”), or rechargeable refillable with e-liquid (“refillable devices”));
  - Smoked < 100 combustible cigarettes in their lifetime^26^ (including zero cigarettes) *and* having not smoked a cigarette in the past three months
  - Never used, or never used than once weekly, any other tobacco/nicotine products.
2. Controls Cohort (N = 257): Satisfied the same criteria (b) and (c) above, and additionally reported having never used, or never fairly regularly used, any e-cigarette/vaping product.

Eligibility for inclusion in each cohort was assessed by a self-administered screening questionnaire that was administered to all individuals who gave informed consent to participate. Efforts were made to match Vapers and Controls in age and sex as closely as possible.

### Procedures

Detailed recruitment and data collection described in the Supplement, and details of survey items have been published previously^25^. Briefly, participants in the Vapers Cohort were asked questions about their historical and current patterns of use of three types of vaping products: disposables, pod/cartridge devices, and refillable devices. For each vaping product category, data were collected on 12 outcomes, as applicable: (1) age of first use; (2) age of initiation of fairly regular use; (3) number of product units used in lifetime; (4) number of product use days in the past 30 days (P30D); (5) length of time (years, months) of fairly regular product use; (6) nicotine content of products use; (7) flavor categories used fairly regularly; (8) number of flavors used fairly regularly; (9) name of favorite flavor used in P30D; (10) number of product units used in P30D; (11) reasons for initiating product use (free text response); and (12) reasons for current product use (free text response).

All participants then completed the Respiratory Symptom Experience Scale (RSES), a validated scale that asks respondents to rate the frequency with which they had experienced five respiratory symptoms in P30D (25). The five symptoms rated were: (1) morning cough with phlegm or mucous; (2) cough frequently throughout the day; (3) shortness of breath that makes it difficult to do normal daily activities; (4) becoming easily winded during normal daily activities; and (5) wheezing or whistling in the chest at times when not exercising or doing other physically strenuous daily activities. Rating for each symptom was made on a 5-point scale: 1 = Never (0 out of the last 30 days); 2 = Rarely (1–5 days); 3 = Occasionally (6–15 days); 4 = Most days (16–29 days); 5 = Every day (all 30 out of the last 30 days). A mean RSES score is calculated by averaging the five item scores. The RSES can be found in the Supplement.

Lastly, questions assessed participants’ sex, country of residence, employment status, highest educational attainment, height, and weight. If needed, surveys were translated to accommodate non-English speakers, with data collected on the language used for the survey to note any nuances lost in translation. As a token of appreciation for participants’ time and contributions, participants were emailed instructions on how to claim a USD $30 Amazon or Take-A-Lot gift card upon survey completion.

### Statistical analyses

Descriptive analyses examined RSES scores and demographic characteristics among Vapers and Control Cohorts. The primary analysis examined differences in RSES score between the Vapers and Control Cohorts using analysis of covariance (ANCOVA) and adjusting for demographic differences (age, sex, education, and employment status). Exploratory follow-up ANCOVAs were conducted on each of the five RSES item scores. *P* values < 0.05 were considered statistically significant.

The secondary objective of this study was to descriptively examine EC use history and EC use patterns among the Vapers Cohort, with respect to history of EC use, past 30-day use patterns, and characteristics of EC products used. Further analyses explored data filtered or stratified by EC device type. All analyses were performed using IBM SPSS version 27.

## Results

### Demographic characteristics

Vapers (N = 491) and Controls (N = 257) were similar on three of the four assessed demographic variables: the majority of each cohort were aged 25–44 years, were employed full-time (≥ 35 h per week), and had some college/university education or has obtained a Bachelor’s degree or higher (Table 1). Vapers were more likely than Controls to be male (54.0% vs 48.6%, respectively).

**Table 1:** Demographic characteristics, by cohort.

Variable	Vaper cohort	Control cohort
	N (%)	N (%)
Total	491 (100.0)	257 (100.0)
Age group		
18–24 years	172 (35.0)	51 (19.8)
25–44 years	308 (62.7)	185 (72.0)
45–64 years	11 (2.2)	21 (8.2)
65 years and older	0 (0.0)	0 (0.0)
Sex		
Male	265 (54.0)	125 (48.6)
Female	215 (44.0)	127 (49.4)
Transgender	3 (0.6)	3 (1.2)
Prefer to not say	7 (1.4)	2 (0.8)
Employment status		
Full-time (≥ 35 h per week)	291 (59.3)	137 (53.3)
Part-time (15–34 h per week)	66 (13.4)	40 (15.6)
Part-time (< 15 h per week)	31 (6.3)	9 (3.5)
Self-employed	44 (9.0)	23 (8.9)
Unemployed	11 (2.2)	7 (2.7)
Retired	1 (0.2)	3 (1.2)
Student	40 (8.1)	34 (13.2)
Stay-at-home parent or spouse	7 (1.4)	4 (1.6)
Prefer not to answer	0 (0.0)	0 (0.0)
Education level		
Less than high school	4 (0.8)	3 (1.2)
High school	65 (13.2)	49 (19.1)
Some college/university	212 (43.2)	90 (35.0)
Bachelor’s degree or higher	208 (42.4)	115 (44.7)
Prefer to not say	2 (0.4)	0 (0.0)

### Respiratory symptoms

**Fig. 2:**
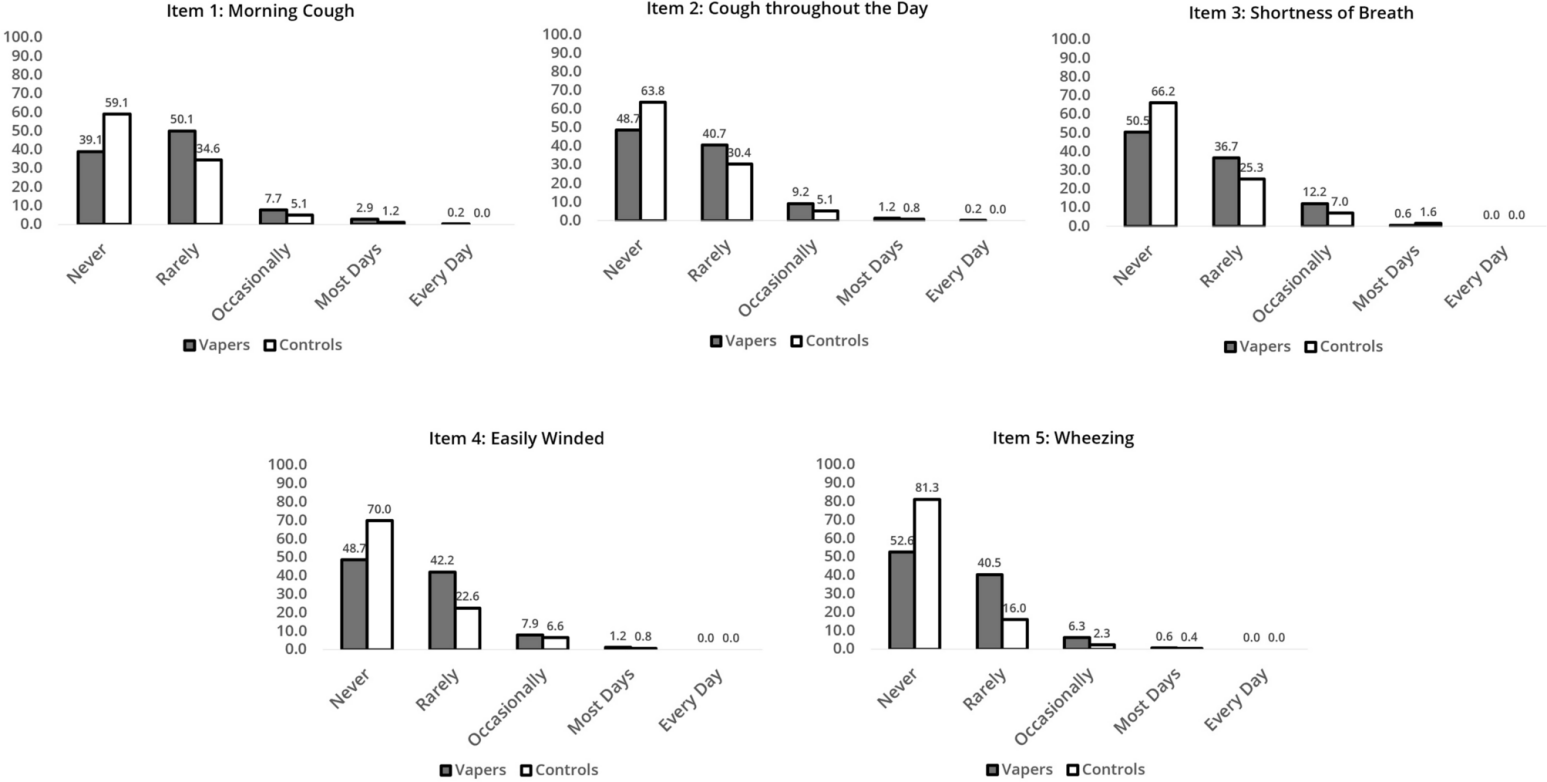
Prevalence and distribution of Respiratory Symptom Evaluation Scale (RSES) score for each RSES item in vaper and control cohorts. The RSES scores range from a minimum of 1 (Never) to a maximum of 5 (Every day), reflecting the frequency of five respiratory symptoms across both study cohorts.

Figure 2 shows the distribution of symptom frequency for each RSES item. Notably, the majority of both Vapers and Control Cohorts (85% or more) reported “never” or “rarely” experiencing each of the five symptoms, which falls below the optimal cut-off point for differentiating participants with (versus without) a diagnosis of respiratory disease^27^. Experiencing occasional symptoms was more common in Vapers than Controls (6.3–12.2% vs. 2.3–7.0%, respectively, across symptoms), but experiencing symptoms most days or everyday was equally rare or non-existent in both groups (0–3.1% for Vapers and 0–1.6% for Controls).

A one-way between-subjects ANCOVA showed a significant difference in mean RSES score between the Vapers and Control Cohorts, after controlling for the effects of age, sex, employment status, and education level, *F* (1, 725) = 18.59, *p* < 0.001, partial *η*^2^ = 0.025. Covariate-adjusted RSES mean scores indicated Vapers (M = 1.61, SE = 0.02) experienced a higher frequency of respiratory symptoms compared to Controls (M = 1.43, SE = 0.03) (mean diff. = 0.18) (Table 2). However, the effect size was small, each cohort’s RSES mean score was close to the minimum scale score of 1 (“never”), and the mean difference between cohorts was below the threshold (0.57) for a clinincally meaningful difference^27^. Moreover, vapers’ RSES score also fell below the optimal cut-off point of 2 (i.e. “rarely”) for differentiating participants with (versus without) a diagnosis of respiratory disease^27^.

**Table 2:** Covariate-adjusted mean score and item scores on the Respiratory Symptom Experience Scale (RSES), by cohort.

Variable	Vaper cohort (N = 479)	Control cohort (N = 252)	*F*	*P*	Partial *η*^2^
	M (SE)	M (SE)			
RSES Mean Score	1.61 (0.02)	1.43 (0.03)	18.59	< 0.001	0.025
Item 1 Score	1.73 (0.03)	1.49 (0.05)	17.12	< 0.001	0.023
Item 2 Score	1.62 (0.03)	1.43 (0.04)	11.90	< 0.001	0.016
Item 3 Score	1.60 (0.03)	1.50 (0.05)	3.34	0.068	0.005
Item 4 Score	1.58 (0.03)	1.45 (0.04)	5.46	0.020	0.007
Item 5 Score	1.51 (0.03)	1.27 (0.04)	24.81	< 0.001	0.033

*N* number, *RSES* Respiratory Symptom Experience Scale, *M* mean, *SE* standard error, *ANCOVA* analysis of covariance.

All ANCOVAs of the main effect of cohort (1 = Vaper Cohort, 2 = Control Cohort) controlled for the effects of age, sex (1 = male, 2 = female), employment status (1 = employed, 2 = not currently employed), and education level (1 = high school or lower, 2 = some college or higher).

Exploratory ANCOVAs conducted separately on each of the five RSES item scores as the dependent variable revealed similarly statistically significant main effects of cohort, with the exception of the ANCOVA conducted on Item 3 score (‘shortness of breath’), which revealed a non-significant covariate-adjusted main effect of cohort ((*F* (1, 725) = 3.34, *p* = 0.068, partial *η*^2^ = 0.005). Vapers reported higher mean scores on each of the five RSES items, with the highest mean score observed on Item 1 (‘morning cough with phlegm or mucous’; M = 1.73) and the highest mean difference score observed on Item 5 (‘wheezing or whistling in the chest while resting’; mean diff. = 0.24).

### History of cigarette smoking and other tobacco/nicotine product use

Though by virtue of incusion in this study, no participants smoked > 100 cigaretes/lifetime; however, the Vapers Cohort more often reported former experimentation with cigarettes (30.8% vs. 12.1% the Control Cohort, Supplement, Table S1). Additionally, a higher proportion of the Vapers Cohort reported use of any other tobacco/nicotine product (18.1% vs. 5.8%).

### EC use patterns

The Vapers Cohort most often used disposable ECs in the past 7 days (63.7%, n = 313), followed by refillable devices (29.1%, n = 143) and pod/cartridge devices (RPFPC, 13.8%, n = 68). A similar preference for disposable ECs was observed when asking about use over different time frames (ever use, ever regular use, and use in P30D) (Supplement, Table S2).

Supplementary Table S3 presents detailed EC use history and product characteristics by primary device type in the past 7 days. Briefly, there was substantial overlap in the use of different EC device types: ~ 13–35% of each group reported also using another device type. There was a wide distribution of use-days in P30D, with ~ 12–19% of the Vapers Cohort using ECs on only 1–5 days, and 12–39% using daily; daily use was more common among those who used refillable devices. Those who used refillable devices also more often reported that their ECs contained nicotine (56%, vs. 28–44% for those who used other device types). While overall, most of the Vapers Cohort reported using ECs for between 1 and 5 years, those who used refillable ECs were more likely to report longer durations of use (25% reported > 5 years, vs. ~ 6–16% of those using different device types). The majority of the Vapers Cohort used multiple flavors in P30D (~ 60–80% across device types used 2+ flavors). Fruit was the most commonly used flavor, used by ~ 70–80% of EC users across device types. Use of tobacco flavor was uncommon (~ 10% or less across device types).

## Discussion

This current VERITAS study provides novel evidence on whether e-cigarette use is uniquely associated with respiratory symptoms, as it is the first such investigation among a large, global cohort of people who do not have an established history of combustible tobacco use. Vapers reported a significantly higher frequency of respiratory symptoms over P30D; however, the difference in RSES score was 0.18, which is approximately one-third as small as the minimal clinically important difference of 0.57 for this scoring^27^. Furthermore, the absolute frequency of these respiratory symptoms among vapers was relatively low, with 20.6% (101 out of 491) of the vaping cohort and 49.4% (127 out of 257) of the control cohort reporting *never* experiencing any symptoms. Yet, comparable proportions of both the vaping (83.3%) and control cohorts (88.4%) reported “never” or “rarely” experiencing symptoms, indicating that more frequenty symptoms were rarely experienced by both groups. While vapers had higher rates of “occasionally” experiencing symptoms, this could be partly due to the transient irritant effects of vaping^28–30^. These observations align with the results of a small, prospective clinical study involving daily vapers who have never smoked. This study reported no regular respiratory symptoms and no significant changes in lung function, inflammation, or structural anomalies as detected in lung CT scans^24^.

Prior research on toxicology and animal models has raised concerns that EC use could pose risks for respiratory illness^31^, though it is not clear whether these findings generalize to human health^32^. Direct evidence from human subjects research is more ambiguous, as, due to ethical considerations, most of this evidence relies on observational data. On one hand, prior observational studies have reported that the prevalence of diagnosed respiratory diseases is higher in EC users than non-users^6–8^. However, since the majority of adult EC users either currently smoke or formerly smoked cigarettes^33,34^ and as cigarette smoking is a well-known cause of respiratory illness^35^, it is highly likely that the apparent association between ECs and respiratory symptoms is confounded by cigarette smoking history^22,35,36^. The current study avoids this limitation by focusing on EC users without a history of established or recent cigarette smoking, and thus provides novel evidence that EC use in the absence of a smoking history is not associated with clinically meaningful increase in frequency of respiratory symptoms. These findings also shed light on the ambiguity in prior research: since EC use is associated with only a small absolute increase in frequency of respiratory symptoms, far below the difference associated with any respiratory diagnoses, it is likely that differences in the likelihood of diagnosed (i.e. clinically relevant) respiratory illness reported in prior studies (6–8) are attributable to significant confounding by unmeasured or uncontrolled effects of individuals’ cigarette smoking history.

With respect to EC use characteristics, the majority of the Vapers Cohort primarily used disposable ECs (64% in P30D), and approximately 30% used refillable devices; pod/cartridge device use was least common, at approximately 15%. Use of multiple types of ECs was common, however, with ~ 13–35% of Vapers reporting using a second EC device type in addition to their primary. The Vapers Cohort had a wide range in frequency of use, with ~ 12–19% (across EC device types) using on only 1–5 days in P30D, and 12–39% reporting daily use. In general, users of refillable devices had use patterns characteristic of heavier use, including more frequent use and having used for a longer duration. Use of non-tobacco flavors was also common across device types—primarily fruit flavors—as was use of multiple flavors (~ 70–80%), especially among users of disposable ECs. This aligns with other recent research showing that disposables are the most common EC device type in recent years^37,38^ and that adult vapers prefer fruit and sweet flavors over traditional cigarette flavors such as tobacco and menthol^37,39^.

The study findings should be interpreted within the context of several limitations. Due to its cross-sectional and observational nature, the current study cannot establish causality between EC use and respiratory symptoms. Some readers may interpret the findings as evidence that vaping increases respiratory symptoms. However, it is equally plausible that individuals with pre-existing respiratory symptoms, due to other factors, are more likely to take up vaping instead of smoking (i.e. reverse causation). There were also other differences between the Vaper and Control Cohorts that could explain the small absolute, but statistically significant, difference in respiratory symptom frequency. The Vaper Cohort, for example, reported slightly more extensive cigarette experimentation (though not rising to the level of established use), and slightly more often use of other tobacco and nicotine products on an occasional basis (versus never). Additionally, there could be other confounding factors that were not measured by the survey (e.g., exposure to environmental or occupational pollutants). Another limitation is that vaping of other substances (e.g., cannabis) was not assessed; this may be important as another possible confounding factor, and could also introduce uncertainty in self-reported vaping behavior (e.g., it is possible that reported use of zero-nicotine EC products could reflect vaping of cannabis or other substances). There was limited information on long-term EC use in the current Vapers Cohort, who predominantly vaped for 5 years or less. Finally, the accuracy of responses may be limited due to recall bias and/or misattribution of EC product characteristics.

This study also has notable strengths: this is a novel study focusing on EC users *without* an established smoking history, which overcomes serious limitations of existing literature and provides preliminary evidence on possible frequency of respiratory symptoms that may be uniquely attributable to EC use. This study is the first, to our knowledge, to focus on this population, and has a large sample drawn from six geopolitical world regions.

In conclusion, this study provides important preliminary evidence that EC use alone (i.e., without a history of established cigarette smoking or recent or regular use of other tobacco or nicotine products) is associated with a small absolute increase in self-reported frequency of respiratory symptoms that is not clinically different from an experience of respiratory symptoms reported by individuals who have never regularly used ECs or any other tobacco or nicotine products. Disposable ECs and use of multiple flavors (predominantly fruit) were the most commonly used product characteristics. This cross-sectional evidence provides a strong basis for mounting longitudinal assessments of intra-individual change in respiratory symptoms among cohorts of EC users with no established smoking or other tobacco or nicotine product use history.

## Supplementary Information

Supplementary Information.

## Data Availability

The raw data presented in this study are not publicly available in order to protect participant confidentiality. However, data are available from the corresponding author Riccardo Polosa upon reasonable request. For any data or questions regarding the study, please contact polosa@unict.it.
