# Supplementary material for: Respiratory symptoms among e-cigarette users without an established smoking history in the VERITAS cohort
Source: Sci Rep. 2024 Nov 18;14:28549. doi: 10.1038/s41598-024-80221-8 (PMC11574031; doi:10.1038/s41598-024-80221-8)
Supplement: Supplementary file 1 — Supplementary Information. [file 41598_2024_80221_MOESM1_ESM.docx]

**SUPPLEMENTARY METHODS**

**Participants**

Sample size calculations and power analysis, detailed below (“Power Analysis to detect differences in respiratory symptoms between two groups”), indicated that, with 75 participants per cohort, the study would have 98% and 89% power to detect a minimal important difference (MID) of 0.57 and 0.40 in RSES scores, respectively. Recruitment targeted enrollment of 750 participants total (Vapers Cohort target n = 500, Control Cohort target n = 250), with approximately 83 participants targeted for recruitment from each of the six world regions.

**Procedure**

A team of recruiters (“VERITAS Ambassadors,” AB, JJCL, AM, KY, MS, ED) identified and pre-screened individuals through various avenues in each world region, including social media channels, WhatsApp groups, email groups, posters in vape shops and universities, referrals from participants, and local vape shop owners (see Supplement for details). The Veritas Ambassadors served as the primary contacts for participants, coordinated by a Project Leader (JZ). Details about roles and responsibilities of the Ambassadors are available at:

<https://veritascohort.coehar.org/wp-content/uploads/2023/08/Ambassador-Job-Description.pdf>

Individuals who expressed an interest in participating were referred by an Ambassador to the study website (<https://veritascohort.coehar.org>) for further information about the study and, if of interest, to complete a brief screener questionnaire to determine eligibility to participate (refer to Appendix). Individuals who did not meet criteria for inclusion in either the Vapers Cohort or the Controls Cohort were terminated from the study and thanked for their time.

Eligible individuals who consented to participate were directed to the study website (<https://veritasuibe.coehar.org>) for a 2-step verification. First, participants registered their email address and, upon receiving a welcome email with a web-link, re-entered the survey website to submit their cell phone number for SMS confirmation. Prior to administering the survey, potential participants underwent a secondary screening via telephone or email to verify eligibility, ensure a comprehensive understanding of the study, and identify the preferred language for the survey.

Participants then completed the main study survey (<https://veritasuibe.coehar.org>) on a device of the participant’s choosing, employing secure online survey tools for centralized data collection.

**POWER ANALYSIS TO DETECT DIFFERENCES IN RESPIRATORY SYMPTOMS BETWEEN TWO GROUPS**

The following graphs show the power to detect a range of group differences (0.30 to 0.80 in absolute differences), for two sample sizes: 60 per group or 75 per group (**Figures S1-S6**). The graphs also use three different estimates of the pooled standard deviation, which affects the posited effect size that is the target of the power analysis (**Figures S1,S2** for SD 0.59**; Figures S3,S4** for SD 0.76**; Figures S5,S6** for SD 0.87).

- The first set is based on the pooled SD from a study by Shiffman et al. 2024 (1) comparing respiratory symptoms between Switchers (i.e., adult smokers who switched completely to JUUL) and Smokers (i.e. adults who continued to smoke) (Pooled SD = 0.59) (**Figures S1,S2)**. This represents the standard way of approaching power analysis.
- The second is more conservative, using the larger of the two SDs from the Smoker group in Shiffman et al. 2024 (1) as an estimate of the pooled SD (SD=0.76) (**Figures S3,S4)**.
- The third uses the pooled SD estimate from the RSES validation study (2), which is larger than either of the other two estimates (pooled SD=0.87) (**Figures S5,S6)**. This is likely much inflated by the fact that the study deliberately over-represented individuals with respiratory disease, including COPD. As a result, this is likely to underestimate power for a study that does not do such over-representation.

NB Note: the graphs differ in the range of the Y-axis (an unalterable feature of the power analysis software), so should not be visually compared, but read out quantitatively against the Y axis.

Conclusion: Initial sample size calculations were based on detecting group differences of 0.57, the estimated MID (Shiffman et al. 2024 [1]). However, it was considered prudent to target a smaller difference of 0.40.  Power analyses considered a range of estimates for the SD, which affects the effect size. It was concluded that samples as small as 75 per group were adequate to achieve power of 89%-98% to detect a difference of 0.40, under a range of assumptions about the SD.

**RSES power analysis**

N=60, Pooled SD = 0.59 (from Shiffman et al. (1))


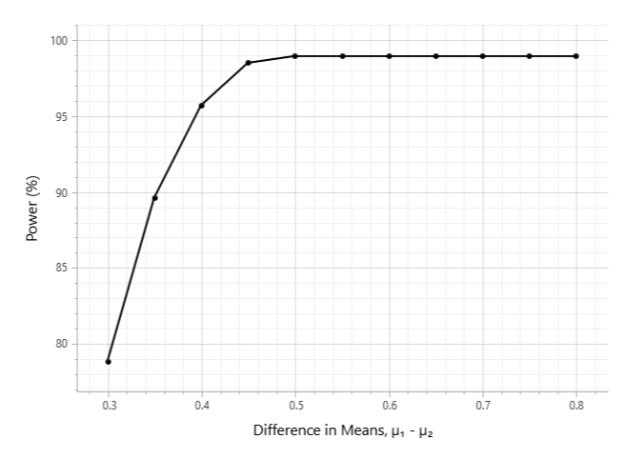


**Figure S1**: *Power Analysis with N=60, Pooled SD = 0.59* (from Shiffman et al. (1)). This graph displays the power to detect differences in respiratory symptoms between two groups with a sample size of 60 per group, using a pooled standard deviation of 0.59 based on Shiffman S, et al. (1). The power estimates range across different group differences from 0.30 to 0.80.

**RSES power analysis**

N=75, Pooled SD = 0.59 (from Shiffman et al. 2024 (1))


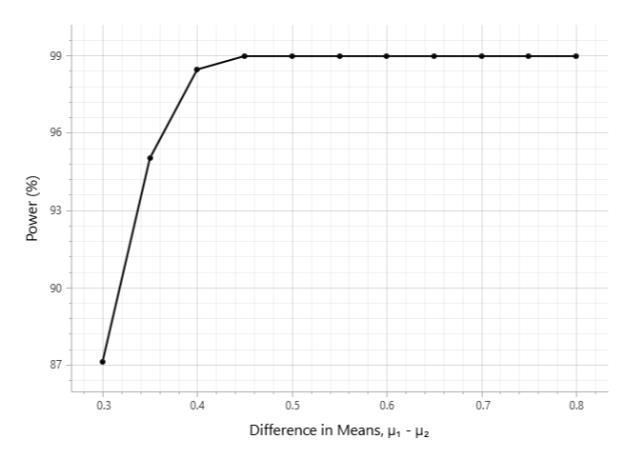


**Figure S2**: *Power Analysis with N=75, Pooled SD = 0.59* (from Shiffman et al. (1)). This graph shows the power to detect differences with a sample size of 75 per group, using the pooled standard deviation of 0.59 from Shiffman S, et al. (1). Power estimates cover group differences from 0.30 to 0.80.

**RSES power analysis**

N=60, larger SD = 0.76 (from Shiffman et al. 2024 (1))


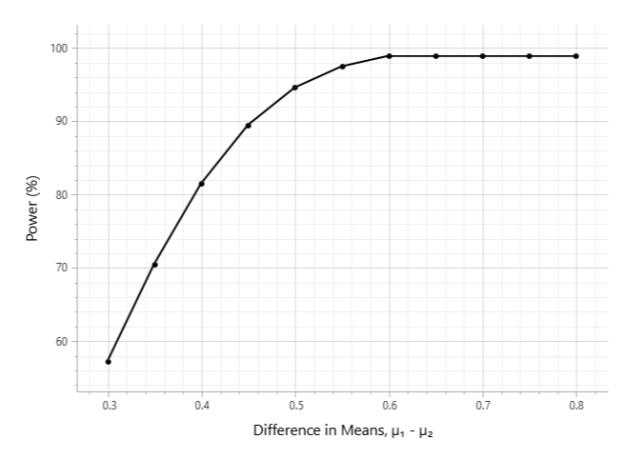


**Figure S3**: *Power Analysis with N=60, Larger SD = 0.76* (from Shiffman et al. (1)). The power analysis for a sample size of 60 per group is presented, using the larger standard deviation (SD = 0.76) from the Shiffman et al. (1). The graph reflects the power to detect absolute differences ranging from 0.30 to 0.80.

**RSES power analysis**

N=75, larger SD = 0.76 (from Shiffman et al. 2024 (1))
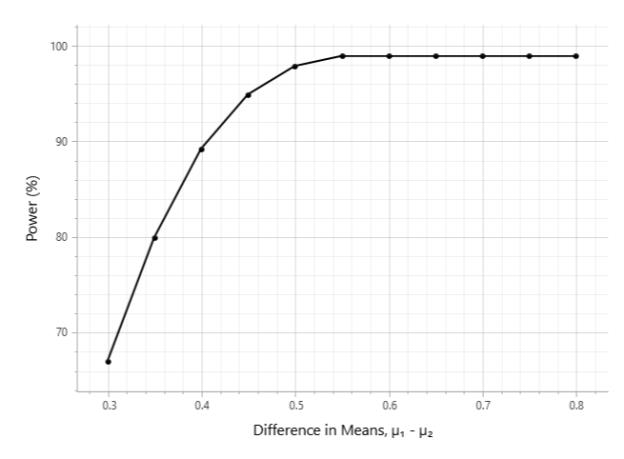


**Figure S4**: *Power Analysis with N=75, Larger SD = 0.76* (from Shiffman et al. (1)). This graph illustrates the power to detect differences with a sample size of 75 per group, using the larger standard deviation (SD = 0.76) from Shiffman et al. (1), across a range of differences from 0.30 to 0.80.

**RSES power analysis**

N=60, Pooled SD = 0.87 (from Shiffman et al. 2023 (2))


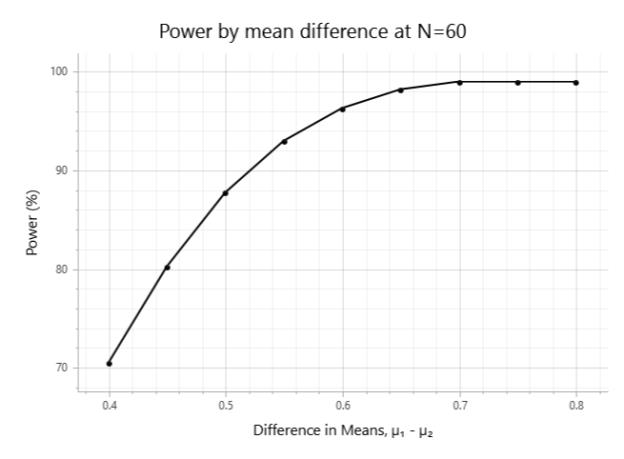


**Figure S5**: *Power Analysis with N=60, Pooled SD = 0.87 (*from Shiffman et al. 2023 (2)*).* This graph presents the power analysis for detecting differences with a sample size of 60 per group, using a pooled SD of 0.87 from Shiffman et al. 2023 (2). The power estimates cover differences from 0.30 to 0.80.

**RSES power analysis**

N=75, Pooled SD = 0.87 (Shiffman et al. 2023 (2))


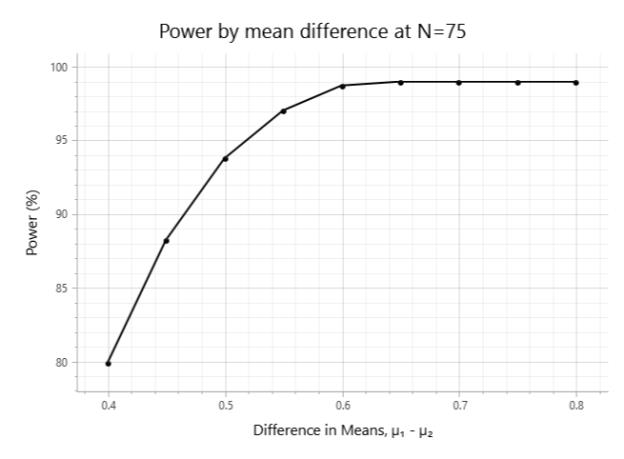


**Figure S6**: *Power Analysis with N=75, Pooled SD = 0.87 (*from Shiffman et al. 2023 (2)*).* The final graph displays the power to detect differences with a sample size of 75 per group, using the pooled SD of 0.87 from Shiffman et al. 2023 (2), across group differences from 0.30 to 0.80.

**REFERENCES**

1. Shiffman S, Oliveri DR, Goldenson NI, Liang Q, Black RA, Mishra S. Comparing Adult Smokers Who Switched to JUUL versus Continuing Smokers: Biomarkers of Exposure and of Potential Harm and Respiratory Symptoms. Nicotine Tob Res. 2024 Mar 22;26(4):494–502.

2. Shiffman S, McCaffrey SA, Hannon MJ, Goldenson NI, Black RA. A new questionnaire to assess respiratory symptoms (The Respiratory Symptom Experience Scale): quantitative psychometric assessment and validation study. JMIR Form Res. 2023;7(1):e44036.

**Pre-Screening Questionnaire**

*This brief questionnaire serves to quickly identify potential participants for VERITAS Cohort study. The recruiter must use it when he/she approaches potential/prospective participants. This can contribute to recruitment success.*

**Questions:**

1. Are you 18 years old or older?
2. Are you currently using electronic cigarettes?
3. Have you been vaping daily for at least three months?
4. Did you smoke any form of combustible tobacco before starting to vape? If so, have you smoked less than 100 cigarettes in your lifetime?
5. Would you be interested in participating in a study? It is a multi-country internet-based survey of adults.
6. If you are interested in participating in the survey, will you accept answering questions regarding age initiation of using vape products; reasons to start using and continuing using vape products; specific types of products used; flavour preferences; nicotine levels; frequency and pattern of use; and self-perceived respiratory health?
7. Do you have an email and phone number to confirm and validate your registration on our platform?

**The Respiratory Symptom Experience Scale (RSES): Questionnaire to assess respiratory symptoms**

In the last 30 days, how many times have you experienced the following?

|  | Never  (0 days of last 30 days) | Rarely  (1-5 days) | Occasionally  (6-15 days) | Almost every day  (16-29 days) | Every day  (all 30 days of the last 30 days) |
| --- | --- | --- | --- | --- | --- |
| Cough in the morning with phlegm or mucus |  |  |  |  |  |
| Cough frequently during the day |  |  |  |  |  |
| My shortness of breath makes it difficult to do normal daily activities, such as climbing a flight of stairs or carrying a heavy object |  |  |  |  |  |
| I am easily out of breath during normal daily activities (for example, doing laundry or carrying groceries) |  |  |  |  |  |
| Sometimes, I hear a hiss or whistle in my chest when I’m not doing exercise or other physically strenuous daily activities (for example, at rest) |  |  |  |  |  |

**Administration and Scoring**

Each item of the RSES is administered reminding participants of the recall period: "In the last 30 days, how many times have you experienced the following?" The RSES items are administered in a fixed order and the order of the answer options is also fixed. It is recommended that researchers use the raw scores of RSES items to calculate a composite. A composite score is calculated by taking the average of the 5 items of the RSES. If an item is missing, no composite should be calculated.

**Table S1.** Use of combustible cigarettes and other nicotine‑containing products, by cohort.

|  | **Vaper Cohort** | **Control Cohort** |
| --- | --- | --- |
| **Variable** | **N (%)** | **N (%)** |
| **Total** | 491 (100.0) | 257 (100.0) |
| **Ever Smoked a Cigarette** |  |  |
| Yes | 151 (30.8) | 31 (12.1) |
| No | 340 (69.2) | 226 (87.9) |
| **N Cigarettes Smoked in Lifetime** |  |  |
| None, not even a puff | 340 (69.2) | 226 (87.9) |
| 1 or more puffs, not a whole cigarette | 35 (7.1) | 20 (7.8) |
| 1 to 10 cigarettes | 51 (10.4) | 4 (1.6) |
| 11 to 20 cigarettes | 31 (6.3) | 1 (0.4) |
| 21 to 50 cigarettes | 19 (3.9) | 4 (1.6) |
| 51 to 99 cigarettes | 15 (3.1) | 2 (0.8) |
| 100 or more | 0 (0.0) | 0 (0.0) |
| Any experimentation (1 puff – 99 cigarettes) | 151 (30.8) | 32 (12.1) |
| **Smoked a Cigarette in Past 3 Months** |  |  |
| Yes | 0 (0.0) | 0 (0.0) |
| No | 491 (100.0) | 257 (100.0) |
| **OTNPs Ever Used** |  |  |
| Traditional cigars | 34 (6.9) | 6 (2.3) |
| Cigarillos or filtered cigars | 11 (2.2) | 5 (1.9) |
| Smoked tobacco in a hookah | 31 (6.3) | 4 (1.6) |
| Smoked tobacco in a pipe | 9 (1.8) | 0 (0.0) |
| Smokeless tobacco (including dip, spit, moist snuff,  pouches, and chewing tobacco) | 3 (0.6) | 1 (0.4) |
| Snus pouches | 8 (1.6) | 3 (1.2) |
| Dissolvable tobacco | 5 (1.0) | 0 (0.0) |
| Heated tobacco products (e.g., iQOS, glo, Pulze) | 19 (3.9) | 0 (0.0) |
| Tobacco-free nicotine pouches | 8 (1.6) | 0 (0.0) |
| One or more of the above | 89 (18.1) | 15 (5.8) |
| None of the above | 402 (81.9) | 242 (94.2) |
| **OTNPs Ever Used Fairly Regularly** |  |  |
| None of the above | 491 (100.0) | 257 (100.0) |
| **OTNPs Used in Past 3 Months** |  |  |
| None of the above | 491 (100.0) | 257 (100.0) |

Abbreviations: N = number; OTNPs = other tobacco and nicotine‑containing products.

**Table S2.** E-cigarette and vaping product use history, by cohort.

|  | **Vaper Cohort** | **Control Cohort** |
| --- | --- | --- |
| **Variable** | **N (%)** | **N (%)** |
| **Total** | 491 (100.0) | 257 (100.0) |
| **Ever Used** |  |  |
| Disposable EC | 355 (72.3) | 9 (3.5) |
| Pod/cartridge EC | 123 (25.1) | 5 (1.9) |
| Refillable EC | 176 (35.8) | 5 (1.9) |
| None of the above | 0 (0.0) | 245 (95.3) |
| **Ever Used Fairly Regularly** |  |  |
| Disposable EC | 318 (64.8) | 0 (0.0) |
| Pod/cartridge EC | 76 (15.5) | 0 (0.0) |
| Refillable EC | 151 (30.8) | 0 (0.0) |
| None of the above | 0 (0.0) | 12 (4.7) |
| **Used in Past 30 Days** |  |  |
| Disposable EC | 314 (64.0) | 0 (0.0) |
| Pod/cartridge EC | 71 (14.5) | 0 (0.0) |
| Refillable EC | 145 (29.5) | 0 (0.0) |
| None of the above | 0 (0.0) | 257 (100.0) |
| **Used in Past 7 Days** |  |  |
| Disposable EC | 313 (63.7) | 0 (0.0) |
| Pod/cartridge EC | 68 (13.8) | 0 (0.0) |
| Refillable EC | 143 (29.1) | 0 (0.0) |
| None of the above | 0 (0.0) | 257 (100.0) |

Abbreviations: N = number; EC = e-cigarettes and vaping products;

**Table S3.** E-cigarette and vaping product use characteristics, by past 7-day EC use group.

|  | | | | **Past 7-Day Users of…** | | | | | | | | | |  |  |  |  |
| --- | --- | --- | --- | --- | --- | --- | --- | --- | --- | --- | --- | --- | --- | --- | --- | --- | --- |
|  | | | | **Disposable ECs** | | | | | **Pod/Cartridge ECs** | | **Rechargeable Refillable ECs** | | |  |  |  |  |
| **Variable** | | | | **N (%)** | | | | | **N (%)** | | **N (%)** | | |  |  |  |  |
| **Total** | | | | 313 (100.0) | | | | | 68 (100.0) | | 143 (100.0) | | |  |  |  |  |
| **Ever Used** | | | |  | | | | |  | |  | | |  |  |  |  |
| Disposable EC | | | | 313 (100.0) | | | | | 21 (30.9) | | 51 (35.7) | | |  |  |  |  |
| Pod/cartridge EC | | | | 42 (13.4) | | | | | 68 (100.0) | | 39 (27.3) | | |  |  |  |  |
| Refillable EC | | | | 42 (13.4) | | | | | 18 (26.5) | | 143 (100.0) | | |  |  |  |  |
| **Ever Used Fairly Regularly** | | | |  | | | | |  | |  | | |  |  |  |  |
| Disposable EC | | | | 313 (100.0) | | | | | 13 (19.1) | | 21 (14.7) | | |  |  |  |  |
| Pod/cartridge EC | | | | 17 (5.4) | | | | | 68 (100.0) | | 12 (8.4) | | |  |  |  |  |
| Refillable EC | | | | 23 (7.3) | | | | | 9 (13.2) | | 143 (100.0) | | |  |  |  |  |
| **Used in the Past 30 Days** | | | |  | | | | |  | |  | | |  |  |  |  |
| Disposable EC | | | | 313 (100.0) | | | | | 12 (17.6) | | 18 (12.6) | | |  |  |  |  |
| Pod/cartridge EC | | | | 13 (4.2) | | | | | 68 (100.0) | | 9 (6.3) | | |  |  |  |  |
| Refillable EC | | | | 19 (6.1) | | | | | 7 (10.3) | | 143 (100.0) | | |  |  |  |  |
| **Used in the Past 7 Days** | | | |  | | | | |  | |  | | |  |  |  |  |
| Disposable EC | | | | 313 (100.0) | | | | | 12 (17.6) | | 17 (11.9) | | |  |  |  |  |
| Pod/cartridge EC | | | | 12 (3.8) | | | | | 68 (100.0) | | 7 (4.9) | | |  |  |  |  |
| Refillable EC | | | | 17 (5.4) | | | | | 7 (10.3) | | 143 (100.0) | | |  |  |  |  |
| **Age of First Use** | | | |  | | | | |  | |  | | |  |  |  |  |
| ≤ 12 years | | | | 0 (0.0) | | | | | 0 (0.0) | | 2 (1.4) | | |  |  |  |  |
| 13-17 years | | | | 18 (5.8) | | | | | 4 (5.9) | | 15 (10.5) | | |  |  |  |  |
| 18-24 years | | | | 238 (76.0) | | | | | 44 (64.7) | | 72 (50.3) | | |  |  |  |  |
| ≥ 25 years | | | | 57 (18.2) | | | | | 20 (29.4) | | 54 (37.8) | | |  |  |  |  |
| **Age First Used Fairly Regularly** | | | |  | | | | |  | |  | | |  |  |  |  |
| ≤ 12 years | | | | 0 (0.0) | | | | | 0 (0.0) | | 0 (0.0) | | |  |  |  |  |
| 13-17 years | | | | 8 (2.6) | | | | | 3 (4.4) | | 6 (4.2) | | |  |  |  |  |
| 18-24 years | | | | 242 (77.3) | | | | | 40 (58.8) | | 75 (52.4) | | |  |  |  |  |
| ≥ 25 years | | | | 63 (20.1) | | | | | 25 (36.8) | | 62 (43.4) | | |  |  |  |  |
| **N Disposable ECs Used in Lifetime** | | | |  | | | | |  | |  | | |  |  |  |  |
| < 100 | | | | 278 (88.8) | | | | | 46 (67.6) | | NA | | |  |  |  |  |
| ≥ 100 | | | | 35 (11.2) | | | | | 22 (32.4) | | NA | | |  |  |  |  |
| **N Days Used a RR EC in Lifetime** | | | |  | | | | |  | |  | | |  |  |  |  |
| < 100 | | | | NA | | | | | NA | | 46 (32.2) | | |  |  |  |  |
| ≥ 100 | | | | NA | | | | | NA | | 97 (67.8) | | |  |  |  |  |
| **N EC Use Days in Past 30 Days** | | | | |  | | | |  | | | | |  |  |  |  |
| 1-5 days | | | | | 39 (12.5) | | | | 13 (19.1) | | | | | 18 (12.6) |  |  |  |
| 6-9 days | | | | | 66 (21.1) | | | | 3 (4.4) | | | | | 6 (4.2) |  |  |  |
| 10-19 days | | | | | 123 (39.3) | | | | 17 (25.0) | | | | | 28 (19.6) |  |  |  |
| 20-29 days | | | | | 49 (15.7) | | | | 22 (32.4) | | | | | 36 (25.2) |  |  |  |
| All 30 days | | | | | 36 (11.5) | | | | 13 (19.1) | | | | | 55 (38.5) |  |  |  |
| **N Years Using Fairly Regularly** | | | | |  | | | |  | | | | |  | |  |  |
| 0-1 years | | | | | 38 (12.1) | | | | 13 (19.1) | | | | | 21 (14.7) | |  |  |
| 1.01-5 years | | | | | 256 (81.8) | | | | 44 (64.7) | | | | | 85 (59.4) | |  |  |
| 5.01-10 years | | | | | 19 (6.1) | | | | 11 (16.2 | | | | | 35 (24.5) | |  |  |
| 10.01-20 years | | | | | 0 (0.0) | | | | 0 (0.0) | | | | | 2 (1.4) | |  |  |
| **ECs Used Now Contain Nicotine** | | | | |  | | | |  | | | | |  | |  |  |
| Yes, always | | | | | 87 (27.8) | | | | 30 (44.1) | | | | | 81 (56.6) | |  |  |
| Yes, most times | | | | | 175 (55.9) | | | | 18 (26.5) | | | | | 31 (21.7) | |  |  |
| Yes, sometimes | | | | | 33 (10.5) | | | | 10 (14.7) | | | | | 24 (16.8) | |  |  |
| No, never | | | | | 18 (5.8) | | | | 10 (14.7) | | | | | 7 (4.9) | |  |  |
| **Nicotine Strength(s) Used in Past 30 Days** | | | | |  | | | |  | | | | |  | |  |  |
| 0mg/mL or 0% | | | | | 27 (8.6) | | | | 12 (17.6) | | | | | 16 (11.2) | |  |  |
| 1-3mg/mL or 0.1-0.3% | | | | | 27 (8.6) | | | | 5 (7.4) | | | | | 39 (27.3) | |  |  |
| 4-6mg/mL or 0.4-0.6% | | | | | 79 (25.2) | | | | 15 (22.1) | | | | | 27 (18.9) | |  |  |
| 7-12mg/mL or 0.7-1.2% | | | | | 103 (32.9) | | | | 13 (19.1) | | | | | 32 (22.4) | |  |  |
| 13-17mg/mL or 1.3-1.7% | | | | | 65 (20.8) | | | | 6 (8.8) | | | | | 12 (8.4) | |  |  |
| 18-24mg/mL or 1.8-2.4% | | | | | 45 (14.4) | | | | 12 (17.6) | | | | | 20 (14.0) | |  |  |
| 25-39mg/mL or 2.5-3.9% | | | | | 18 (5.8) | | | | 9 (13.2) | | | | | 12 (8.4) | |  |  |
| 40-49mg/mL or 4.0-4.9% | | | | | 17 (5.4) | | | | 7 (10.3) | | | | | 7 (4.9) | |  |  |
| 50-59mg/mL or 5.0-5.9% | | | | | 14 (4.5) | | | | 4 (5.9) | | | | | 6 (4.2) | |  |  |
| 60+mg/mL or 6.0+% | | | | | 11 (3.5) | | | | 1 (1.5) | | | | | 6 (4.2) | |  |  |
| Don’t Know | | | | | 11 (3.5) | | | | 1 (.5) | | | | | 6 (4.2) | |  |  |
| **EC Flavour(s) Used in Past 30 Days** | | | | |  | | | |  | | | | |  | | |  |
| Tobacco | | | | | 12 (3.8) | | | | 7 (10.3) | | | | | 10 (7.0) | | |  |
| Menthol | | | | | 29 (9.3) | | | | 15 (22.1) | | | | | 28 (19.6) | | |  |
| Mint | | | | | 147 (47.0) | | | | 20 (29.4) | | | | | 50 (35.0) | | |  |
| Tropical Fruit | | | | | 177 (56.5) | | | | 22 (32.4) | | | | | 72 (50.3) | | |  |
| Citrus Fruit | | | | | 125 (39.9) | | | | 14 (20.6) | | | | | 48 (33.6) | | |  |
| Berries | | | | | 105 (33.5) | | | | 28 (41.2) | | | | | 67 (46.9) | | |  |
| Other Fruit | | | | | 38 (12.1) | | | | 8 (11.8) | | | | | 51 (35.7) | | |  |
| Dessert | | | | | 15 (4.8) | | | | 7 (10.3) | | | | | 27 (18.9) | | |  |
| Candy | | | | | 20 (6.4) | | | | 5 (7.4) | | | | | 16 (11.2) | | |  |
| Other Sweets | | | | | 10 (3.2) | | | | 2 (2.9) | | | | | 20 (14.0) | | |  |
| Nuts | | | | | 3 (1.0) | | | | 1 (1.5) | | | | | 7 (4.9) | | |  |
| Spices | | | | | 2 (0.6) | | | | 0 (0.0) | | | | | 4 (2.8) | | |  |
| Coffee/Tea | | | | | 11 (3.5) | | | | 1 (1.5) | | | | | 12 (8.4) | | |  |
| Alcohol | | | | | 9 (2.9) | | | | 2 (2.9) | | | | | 3 (2.1) | | |  |
| Other Beverages | | | | | 15 (4.8) | | | | 1 (1.5) | | | | | 6 (4.2) | | |  |
| Unflavoured | | | | | 2 (0.6) | | | | 1 (1.5) | | | | | 3 (2.1) | | |  |
| Other Flavour | | | | | 3 (1.0) | | | | 0 (0.0) | | | | | 4 (2.8) | | |  |
| **N Different EC Flavours Used in Past 30 Days** | | | | |  | | | |  | | | | |  | | |  |
| 1 | | | | | 70 (22.4) | | | | 26 (38.2) | | | | | 44 (30.8) | | |  |
| 2 | | | | | 160 (51.1) | | | | 25 (36.8) | | | | | 44 (30.8) | | |  |
| 3 | | | | | 48 (15.3) | | | | 10 (14.7) | | | | | 28 (19.6) | | |  |
| 4 | | | | | 18 (5.8) | | | | 3 (4.4) | | | | | 10 (7.0) | | |  |
| 5 or more | | | | | 17 (5.4) | | | | 4 (5.9) | | | | | 17 (11.9) | | |  |
| **N Disposable ECs used in Past 30 Days** | | | | |  | | | |  | | | | |  | | | |
| 1 | | | | | 30 (9.6) | | | | NA | | | | | NA | | | |
| 2 | | | | | 62 (19.8) | | | | NA | | | | | NA | | | |
| 3 | | | | | 51 (16.3) | | | | NA | | | | | NA | | | |
| 4 | | | | | 76 (24.3) | | | | NA | | | | | NA | | | |
| 5 | | | | | 36 (11.5) | | | | NA | | | | | NA | | | |
| 6 | | | | | 14 (4.5) | | | | NA | | | | | NA | | | |
| 7 or more | | | | | 44 (14.1) | | | | NA | | | | | NA | | | |
| **N Pod/Cartridge ECs used in Past 30 Days** | | | | |  | | | |  | | | | |  | | | |
| 1 | | | | | NA | | | | 8 (11.8) | | | | | NA | | | |
| 2 | | | | | NA | | | | 14 (20.6) | | | | | NA | | | |
| 3 | | | | | NA | | | | 14 (20.6) | | | | | NA | | | |
| 4 | | | | | NA | | | | 6 (8.8) | | | | | NA | | | |
| 5 | | | | | NA | | | | 7 (10.3) | | | | | NA | | | |
| 6 | | | | | NA | | | | 3 (4.4) | | | | | NA | | | |
| 7 or more | | | | | NA | | | | 16 (23.5) | | | | | NA | | | |
| **N Millilitres of E-Liquid Used in Past 30 Days** | | | | |  | | | |  | | | | |  | | | |
| < 30 | | | | | NA | | | | NA | | | | | 9 (6.3) | | | |
| 30 to < 60 | | | | | NA | | | | NA | | | | | 23 (16.1) | | | |
| 60 to < 90 | | | | | NA | | | | NA | | | | | 31 (21.7) | | | |
| 90 to <120 | | | | | NA | | | | NA | | | | | 6 (4.2) | | | |
| 120 to < 150 | | | | | NA | | | | NA | | | | | 22 (15.4) | | | |
| 150 to < 180 | | | | | NA | | | | NA | | | | | 8 (5.6) | | | |
| 180 to < 210 | | | | | NA | | | | NA | | | | | 2 (1.4) | | | |
| 210 to < 240 | | | | | NA | | | | NA | | | | | 10 (7.0) | | | |
| 240 to < 270 | | | | | NA | | | | NA | | | | | 16 (11.2) | | | |
| 270 or more | | | | | NA | | | | NA | | | | | 16 (11.2) | | | |

Abbreviations: N = number; EC = e-cigarettes and vaping products; RR = rechargeable, refillable; mg/mL = milligrams of nicotine per millilitre; NA = not applicable.

In response to the reviewer’s comment, we conducted an exploratory one‑way between‑subjects ANCOVA of the effect of lifetime number of cigarettes smoked (0 vs. 1‑99) on mean score on the Respiratory Symptom Experience Scale (RSES) within the Vaper Cohort when controlling for the effects of age, sex, employment status, and education level. A significant main effect of lifetime number of cigarettes smoked was found, with covariate‑adjusted RSES mean scores indicating that vapers who had never smoked a cigarette (M = 1.66, SE = 0.28) reported a higher frequency of respiratory symptoms compared to vapers who had smoked 1‑99 cigarettes in their lifetime (M = 1.54, SE = 0.43) (mean diff. = 0.12) (Table S4). However, as was the observed for the main ANCOVA conducted on the effect of cohort on RSES score (reported in the manuscript), the effect size was small, each sub-group’s RSES mean score was close to the minimum scale score of 1, and the mean difference between cohorts was lower than 0.5, the level established by the scale creators as being the minimum difference that can be interpreted as reflecting a meaningful change or difference in the frequency of respiratory symptoms. Additionally, an exploratory ANCOVA of the effect of lifetime number of cigarettes smoked (0 vs. 1‑99) on mean score on the RSES within the Control Cohort showed no significant main effect of lifetime number of cigarettes smoked. The results of these exploratory ANCOVAs therefore neither change nor meaningfully add to the results of the main ANCOVAs of the effect of cohort on RSES score reported in the manuscript.

Importantly, the fact that these exploratory analyses of the effect of lifetime cigarette consumption (0 vs. 1‑99) showed no change in results suggests that the recruitment criteria used for the VERITAS cohort remain valid.

Table S4. Covariate‑adjusted mean score and item scores on the Respiratory Symptom Experience Scale (RSES), by number of cigarettes smoked in lifetime within cohort.

|  | **N Cigarettes Smoked in Lifetime** | |  |  |  |
| --- | --- | --- | --- | --- | --- |
| ***Vapers Cohort*** | **0.1-99**  **(N = 142)** | **0**  **(N = 337)** |  |  |  |
| **Variable** | **M (SE)** | **M (SE)** | ***F*** | ***p*** | **partial *η*^2^** |
| **RSES Mean Score** | 1.54 (0.43) | 1.66 (0.28) | 5.30 | 0.022 | 0.011 |
| Item 1 Score | 1.73 (0.39) | 1.73 (0.06) | 0.00 | 0.973 | 0.000 |
| Item 2 Score | 1.61 (0.06) | 1.63 (0.04) | 0.05 | 0.821 | 0.000 |
| Item 3 Score | 1.69 (0.04) | 1.49 (0.06) | 7.45 | 0.007 | 0.015 |
| Item 4 Score | 1.67 (0.04) | 1.49 (0.06) | 6.51 | 0.011 | 0.014 |
| Item 5 Score | 1.60 (0.03) | 1.40 (0.05) | 10.24 | 0.001 | 0.021 |
| ***Control Cohort*** | **0.1-99**  **(N = 31)** | **0**  **(N = 221)** |  |  |  |
| **Variable** | **M (SE)** | **M (SE)** | ***F*** | ***p*** | **partial *η*^2^** |
| **RSES Mean Score** | 1.50 (0.09) | 1.38 (0.03) | 1.46 | 0.228 | 0.006 |
| Item 1 Score | 1.52 (0.12) | 1.49 (0.04) | 0.09 | 0.771 | 0.000 |
| Item 2 Score | 1.47 (0.11) | 1.42 (0.42) | 0.13 | 0.717 | 0.001 |
| Item 3 Score | 1.62 (0.13) | 1.43 (0.05) | 2.09 | 0.150 | 0.008 |
| Item 4 Score | 1.55 (0.12) | 1.36 (0.04) | 2.32 | 0.129 | 0.009 |
| Item 5 Score | 1.34 (0.09) | 1.21 (0.03) | 1.88 | 0.172 | 0.008 |

Abbreviations: N = number; RSES = Respiratory Symptom Experience Scale; M = mean; SE = standard error; ANCOVA = analysis of covariance.

Note: All ANCOVAs of the main effect of ‘number of cigarettes smoked in lifetime’ (1= 0.1-99, 2 = 0 (zero)) controlled for the effects of age, sex (1 = male, 2 = female), employment status (1 = employed, 2 = not currently employed), and education level (1 = high school or lower, 2 = some college or higher).
